# Stigma in Parkinson's disease: Placing it outside the body

**DOI:** 10.1016/j.clinsp.2022.100008

**Published:** 2022-02-13

**Authors:** Tomás de la Rosa, Fúlvio Alexandre Scorza

**Affiliations:** Neurology Department, Escola Paulista de Medicina, Universidade Federal de São Paulo, São Paulo, SP, Brazil

**Keywords:** PD, Parkinson's Disease, ADL, Activities of Daily Living

## Stigma in Parkinson's disease

Parkinson's Disease (PD) has traditionally been characterized by its motor symptoms (e.g., bradykinesia, rigidity, and tremor),^1^ however the disease burden also comprises other non-motor symptoms^2^ and psycho-social problems that can negatively impact patients Health-Related Quality of Life.^3^ A significant one is a stigma which both PD and caregivers experience due to their condition. Stigma could be defined as an attribute implying a discredit of the individual who is considered “bad, weak or dangerous”, reducing it to a representation from a whole and usual person to a tainted one.^4^ As a matter, more than 50% of patients with PD conceal their diagnosis,^5^ trying to mask some of their clinical symptoms[6] or even avoid appearing in public.^7^ This stigma emanates from the interplay between patient and environment, where stigmas place the burden on the stigmatized subject.^8^^,^^9^ Like any other neuropsychiatric disease, PD is a categorization tool used by physicians and researchers for a better understanding of this phenomenon and the development of effective therapies and care. However, this tool is embedded in social, cultural, and political dimensions, which directly affect the construction of stigma against these populations. Efforts are made by researchers and clinicians in order to minimize the effect stigma has on patient wellbeing and quality of life, often analyzing how some disease characteristics like severity of motor symptoms or emotional disorders affect this situation. Despite the direct relation patient functional and bodily states may have with stigma, the authors should consider this socio-cultural component intrinsic to this phenomenon in order to accurately approach a solution.

Clinical symptoms observed in PD patients can lead to communicative and social disruptions,^10^ especially those symptoms related to emotion expression and recognition like facial masking, constituting what has been named as ‘social symptoms of PD’,^11^ which largely contribute to stigma experience. In this line, clinical research has also explored how stigma could be predicted from disease characteristics like depression,^12^^,^^13^ low scores in Activities of Daily Living (ADL),^12^^,^^14^ or severe motor symptoms.^13^^,^^15^ These associations between clinical symptoms and stigma are usually followed by the logical conclusion that ameliorating those symptoms is the path to take to effectively reduce stigma in PD patients, further suggesting the collection of more biomedical variables with the potential to emerge as predictors of stigma. Besides the evident impact motor symptoms improvement could have on experienced stigma in PD patients, studying stigma solely through the analysis of symptoms and functional capacities of patients places the burden of stigma on the bodies of patients, largely neglecting the socio-cultural dimension intrinsic to stigma phenomenon.^8^ Authors are often aware of this conflict, as they state the importance of this socio-cultural dimension, while at the same time, the variables explored relate almost exclusively to patients’ bodies, and the socio-cultural aspect remains unexplored. Despite the focus on clinical symptoms associated with stigma in PD, these studies also reported differences in the few social variables they collected like gender[13] or age,^13^^,^^14^ reinforcing the standpoint of stigma as socio-cultural informed. Results also showed how emotional disorders were robust predictors of stigma,^12^^,^^13^ which are largely affected by social discrimination.^16^.

This gap between the socio-cultural dimension of stigma and the bodily states of patients reflects the relation between epistemology and ontology within biomedical research and practice, showing how the production of knowledge could be influenced by socio-cultural contexts. Therefore, the authors need approaches that tackle this complexity, further exploring this aspect of stigma. The review of Maffoni et al.^17^ try to describe a new understanding of stigma from an intercultural and social viewpoint, moving to a patient-centered approach that contextualizes clinical symptoms within a broader dimension of socio-cultural interactions. This kind of understanding of stigma in PD is present in other studies,^11^ where authors also stress the importance of properly identifying stigma when it is invisible to physicians.^6^ Henry et al.^18^ reported differences in stigma between Mexico and USA patients and caregivers, showing potential cultural differences of stigma. The authors share with these authors the notion of stigma as a subjective symptom, which, besides its relationship with clinical symptoms, emerges mainly from the interaction between individuals and society as a whole.

Clinicians and researchers are also social actors embedded in socio-cultural environments regarding the biomedical care they deliver. In addition to the treatment of motor and non-motor symptoms, which largely impact patients' wellbeing, they could also address the social and political dimensions encompassing diseases like PD, aiming to alter current understandings and create responses from a clearer view of patient's experiences.^19^ If stigma is fundamentally a social-based issue, why put the focus on biomedical variables regarding the patients’ body, when it would be more relevant to explore factors in direct association with social discrimination and stigma like socioeconomic status, prior experience of trauma, accessibility to healthcare specialists or access to caregiving.^20^ This way, the authors would be placing the burden of the stigma where it belongs, outside patients' bodies, both in clinical practice and in research.

## Conflicts of interest

The authors declare no conflicts of interest.

## References

[1] Poewe W, Seppi K, Tanner CM, Halliday GM, Brundin P, Volkmann J (2017). Parkinson disease. Nat Rev Dis Primers.

[2] Marras C, Chaudhuri KR. (2016). Nonmotor features of Parkinson's disease subtypes. Mov Disord.

[3] Ma HI, Saint-Hilaire M, Thomas CA, Tickle-Degnen L. (2016). Stigma as a key determinant of health-related quality of life in Parkinson's disease. Qual Life Res.

[4] Goffman E. Stigma: Notes on the management of spoiled identity. 1986.

[5] Werner P, Korczyn AD. (2010). Lay persons’ beliefs and knowledge about Parkinson's disease: prevalence and socio-demographic correlates. Parkinsonism Relat Disord.

[6] Hermanns M. (2013). The invisible and visible stigmatization of Parkinson's disease. J Am Assoc Nurse Pract.

[7] Nijhof G. (1995). Parkinson's disease as a problem of shame in public appearance. Sociol Health Illn.

[8] Jones EE. Social stigma: the psychology of marked relationships. 1984.

[9] Major B, O'Brien LT. (2005). The social psychology of stigma. Annu Rev Psychol.

[10] Soleimani MA, Negarandeh R, Bastani F, Greysen R. (2014). Disrupted social connectedness in people with Parkinson's disease. Br J Community Nurs.

[11] Prenger MTM, Madray R, Van Hedger K, Anello M, MacDonald PA. (2020). Social symptoms of Parkinson's disease. Parkinsons Dis.

[12] Salazar RD, Weizenbaum E, Ellis TD, Earhart GM, Ford MP, Dibble LE (2019). Predictors of self-perceived stigma in Parkinson's disease. Parkinsonism Relat Disord.

[13] Hou M, Mao X, Hou X, Li K. (2021). Stigma and associated correlates of elderly patients with Parkinson's disease. Front Psychiatry.

[14] da Silva AG, Leal VP, da Silva PR, Freitas FC, Linhares MN, Walz R (2020). Difficulties in activities of daily living are associated with stigma in patients with Parkinson's disease who are candidates for deep brain stimulation. Braz J Psychiatry.

[15] Gunnery S, Saint-Hilaire M, Thomas C, Tickle-Degnen L. (2015). Emerging evidence for facial muscle action as a predictor of experienced stigma in Parkinson's disease. Arch Phys Med Rehabil.

[16] Alvarez-Galvez J, Rojas-Garcia A. (2019). Measuring the impact of multiple discrimination on depression in Europe. BMC Public Health.

[17] Maffoni M, Giardini A, Pierobon A, Ferrazzoli D, Frazzitta G. (2017). Stigma experienced by Parkinson's disease patients: a descriptive review of qualitative studies. Parkinsons Dis.

[18] Henry RS, Perrin PB, Lageman SK, Villaseñor T, Cariello AN, Pugh M (2021). Parkinson's symptoms and caregiver affiliate stigma: a multinational study. Curr Alzheimer Res.

[19] Valcarenghi RV, Alvarez AM, Santos SSC, Siewert JS, Nunes SFL, Tomasi AVR. (2018). The daily lives of people with Parkinson's disease. Rev Bras Enferm.

[20] Nugent C, Rosato M, Hughes L, Leavey G. (2021). Risk factors associated with experienced stigma among people diagnosed with mental ill-health: a cross-sectional study. Psychiatr Q.

